# OP10 Can Manufacturers Leverage Social Media Listening Studies To Generate Payer-Relevant Data For Health Technology Assessments?

**DOI:** 10.1017/S026646232510069X

**Published:** 2025-12-29

**Authors:** Ally Robert, Smarth Lakhanpal

## Abstract

**Introduction:**

Social media listening studies (SMLS) analyze online dialogue from patients, caregivers, and clinicians to gain insights into the lived experience of disease including treatment efficacy and safety, and unmet needs. This analysis aims to understand whether SMLS is a feasible method for generating real-world data that can shape pivotal trial design or supplement trial data for health technology assessment (HTA).

**Methods:**

A targeted literature search was conducted on PubMed to identify articles published in English that describe and report the findings of SMLS specifically investigating health conditions and diseases. No limits were placed on date of publication or geographical scope. Information was extracted from these papers and used to map concepts and themes covered in SMLS to key domains of HTA submission dossiers in the UK, France, and Germany, as well as the upcoming joint clinical assessments (JCA). This analysis was used to determine the extent to which SMLS can be used to generate payer-relevant data.

**Results:**

A total of 19 papers were included in this analysis. All studies used a retrospective approach, and the majority (84%) analyzed data from more than one market. The most common markets included were the UK (74%), USA (58%), France, and Germany (53%). Thirty-two percent included only patient conversations; 47 percent included conversations from patients, caregivers, family, and friends; and 21 percent included physicians. The most common themes covered were quality of life (89%), burden of disease or symptomology (79%), and treatment patterns (79%), which align closest to the dossier templates for HAS (French National Authority for Health) and the JCA.

**Conclusions:**

All studies in this analysis included data that would be relevant for HTA submission, and the majority provided data that would speak to multiple payer concerns. While there are no published guidelines for leveraging SMLS for HTA, this analysis indicated that this methodology could potentially be used alongside trial data to highlight the patient experience within reimbursement submissions.

